# Maternal supplementation with phytogenic additives influenced the faecal microbiota and reproductive potential in sows

**DOI:** 10.1186/s13568-021-01268-8

**Published:** 2021-07-15

**Authors:** Tanya L. Nowland, Dragana Stanley, Roy N. Kirkwood, Valeria A. Torok, Yadav S. Bajagai, Neil J. Gannon, Kate J. Plush

**Affiliations:** 1 School of Animal and Veterinary Sciences, The University of Adelaide, 5371 Roseworthy, SA Australia; 2 Institute for Future Farming Systems, Central Queensland University, 4701 Rockhampton, QLD Australia; 3 Food Sciences, South Australian Research and Development Institute, SA 5064 Urrbrae, Australia; 4 BIOMIN Pte. Ltd, 159741 Singapore, Singapore; 5 SunPork Group, QLD 4009 Eagle Farm, Australia

**Keywords:** Bacteria, Gut health, Production, Pig, PFA

## Abstract

Sows undergo physiological stress during gestation and lactation, potentially leading to enteric dysbiosis and reduced reproductive potential. Phytogenic additives (PFs) may improve performance via their antioxidant, anti-inflammatory and antimicrobial properties. This study determined whether the provision of a gestation/lactation diet containing PAs would alter the gastrointestinal microbiota of sows and their piglets, and improve performance. Sows received a commercial diet throughout gestation and lactation (CTR; n = 64), a commercial diet throughout gestation and a diet containing PAs in lactation (CTR-PA; n = 63) or a commercial diet containing PAs in gestation and lactation (PA; n = 90). Sows were weighed and backfat recorded after mating and at entry and exit from the farrowing house and piglets were weighed on days 1 and 21 of life. Faecal samples collected from sows at farrowing house entry and piglets at 21 and 35 d were subjected to 16 S rRNA gene amplicon analysis. The addition of PAs to sow diets resulted in more piglets born (*P* = 0.03), however, it did not improve the number of liveborn piglets (*P* = 0.14). There were no differences in sow weight, P2 backfat depth or lactation feed intake observed. PAs had no effect on piglet weight or survival to weaning but did alter the faecal microbiota of sows, and this change was observed in piglets at 21 and 35 d. PA supplementation to sows has the potential to increase litter size, while also potentially influencing gastrointestinal tract health of the sow and piglets reared.

**Supplementary Information:**

The online version contains supplementary material available at 10.1186/s13568-021-01268-8.

## Key points

- Number of piglets born was increased through phytogenic supplementation to sows.
- *Oscillospira, Roseburia* and *Ruminococcus* were increased in sows fed phytogenics.
- Sow phytogenic supplementation increased *Faecalibacterium* in piglets post-weaning.

## Introduction

Gestation and lactation are both times of high physiological stress for sows. Gestation involves the partitioning of nutrients for the development of multiple foetuses, while lactation has great demands on sows as they produce enough milk to feed their litter. Often due to this highly taxing process, sows lose from 5 to 20% of their body weight (Thaker and Bilkei 2005). Stress can also decrease food intake and induce enteric dysbiosis in pigs, which can cause suboptimal digestion and poor nutrient utilisation and negatively affect intestinal health (Gresse et al. 2017). Impaired nutrient intake and utilisation increases weight loss and can have a negative effect on their ability to rear their litter and to return to oestrus after their litter is weaned (Thaker and Bilkei 2005). Additionally, sows undergo large shifts in the gastrointestinal tract (GIT) microbiota throughout this time (Gaukroger et al. 2020) and exhibit metabolic syndrome in late gestation and early lactation (Cheng et al. 2018). Nutritional interventions may improve sow rearing ability and reduce negative effects on their health.

Phytogenics are a group of natural flavour and sensory compounds derived from plants and include herbs, spices and essential oils (Windisch et al. 2008). When added to feed, they improve animal performance via three main mechanisms; flavour properties which enhance feed intake, biological activity that aids digestion, and improving GIT health via modulation of the GIT microbiota (Windisch et al. 2008; Murugesan et al. 2015). The proposed drivers for these influences on performance are the antioxidant, anti-inflammatory and antimicrobial properties they exhibit (Windisch et al. 2008).

Recent work in pigs demonstrated that the phytogenic additive (PA) which includes a combination of essential oils, maintained finisher performance when protein and energy specifications in the diet were reduced, and improved performance when dietary specifications were maintained (Walker et al. 2019). However, there is little published data on the effect of phytogenics on sow reproduction or the GIT microbiota. Additionally, given that piglets are raised within a farrowing crate in direct contact with their sow, it is likely that the establishment of the piglet GIT microbiota is dependent on contact with their mother. We aimed to determine whether the provision of gestation and/or lactation diets containing PAs would alter the GIT microbiota of sows, and thus that of their piglets, and so improve performance. It was hypothesised that (1) the provision of a diet containing PAs during gestation would increase litter birth weight, and when fed during lactation would increase sow feed intake and lactation performance; (2) the provision of a gestation/lactation diet containing PAs would alter the GIT microbiota of the sow, with this change transmitted to their piglets causing a shift in piglet GIT microbiota and improvements in their growth and survival.

## Materials and methods

### Sow housing and feeding management

After mating, 351 sows (parity 2 to 4) were allocated to one of six identical, naturally ventilated gestation pens (1.8 m^2^ per sow) based on mating date and parity. The pens had partially slatted concrete flooring with eight drinkers per pen. Sows were housed in groups of ~ 60 and fed via electronic sow feeders (ESF; MPS Agri Ltd, Suffolk, UK). The electronic sow feeders enabled the feeding of two separate diets to pigs within the same pen. Sows were allowed 2.2 kg/day of a commercial gestation diet formulated to provide 13.0 MJ DE/kg, 13.1 % total protein and 0.55 % standardised ileal digestible (SID) lysine unless their P2 backfat depth (P2; 65 mm off the midline at the last rib curve) at breeding was < 14 mm, when the allowance was increased to 2.8 kg/day for the first 30 days and then subsequently reduced to 2.2 kg/day until moved to farrowing accommodation. Pregnancy confirmation was performed by B-mode ultrasonography at 35 d and 70 d post-breeding and any non-pregnant sows removed from the pen.

At 5.7 ± 0.4 d prior to their calculated farrowing date, sows were moved into naturally ventilated farrowing accommodations and housed in individual farrowing crates (1.8 × 2.4 m). Each farrowing crate contained its own lamp heated creep area for the piglets and two water nipples for the sow and one for the piglets. Prior to farrowing, sows were fed 2.4 kg/d of a commercial lactation diet formulated to provide 14 MJ DE/kg, 17.3 % total protein and 0.84 % SID lysine. After farrowing, sows were fed the lactation diet to-appetite up to 16 kg/d delivered in two meals until weaning at 22.4 ± 0.1 d.

At the time of breeding, sows were assigned to one of three dietary treatments to have equal parity distributions, previous litter size and wean-to-serve intervals. Treatments were:

1. CTR: fed a commercial diet in gestation and lactation (n = 64).
2. PA: fed a commercial diet containing a phytogenic additive (PA) (700 g/t) in gestation and lactation (n = 90).
3. CTR-PA: fed a commercial diet in gestation and a diet containing a PA (700 g/t) in lactation (n = 63).

Base diet specifications used are outlined in Additional file 1: Table S1. The PA used throughout the study was Digestarom® DC Xcel 1000 provided by BIOMIN (BIOMIN Animal Nutrition GmbH, Getzersdorf, Austria) and contained a proprietary mix of essential oil extracts and herbs with menthol, carvacrol, carvone as major bioactive compounds. 700 g of the proprietary mix was added to each tonne of base diet via micro dispenser. The proprietary mix was microencapsulated to ensure heat stability during pelleting.

### Data recorded

All sows were weighed and their P2 backfat depths recorded at entry into the gestation housing and on entry and exit from the farrowing house. Sow feed intakes in the farrowing house were measured by weighing all leftover feed and all new feed into the feeder when sows were fed twice daily. On the day of farrowing, the total born and live-born litter sizes and individual birth weights were recorded. At farrowing, two live female focal piglets per litter were tagged to allow individual identification. At 13 h and within 24 h of farrowing, fostering occurred within treatment based on the sows rearing capacity (functional teat number) and all piglet movement was noted. Litter weight was recorded on day 1 and 21 of lactation. Individual piglet weights on day 1 were used to determine the total litter weight, minimum and maximum piglet weight and the percentage of piglets within the litter weighing less than 1.1 kg. All mortalities and removals for ill thrift were recorded, as were the number of pigs weaned per sow and the time from weaning to mating. Faecal samples were collected from sows at weighing prior to farrowing house entry and from tagged focal piglets at 21 (prior to weaning) and at 35 days of age (~ 2 weeks postweaning). The focal piglets were individually weighed at 21 and at 35 days of age. Faeces were placed on ice immediately and stored at − 80^o^C within 4 h of collection.

### DNA extraction and 16 S rRNA amplicon analysis

Approximately 0.2 g from each sample was used for the DNA extraction using the modified repeated bead beating plus column method (Yu and Morrison 2004) and the quantity of DNA was estimated using a NanoDrop spectrophotometer (ThermoFisher Scientific, Massachusetts, USA).

The forward and reverse primers used for amplification of the V3-V4 region of the 16 S rRNA gene were: ACTCCTACGGGAGGCAGCAG and GGACTACHVGGGTWTCTAAT, respectively. The 16 S rRNA gene amplicon sequencing library was prepared by amplifying the V3-V4 region of the gene with the primers containing linker sequences, index sequences and heterogenicity spacers (Fadrosh et al. 2014). The amplified amplicon library was cleaned up using AMPure XP clean up kit (Beckman Coulter, Lane Cost West, NSW, Australia). Sequencing was conducted on the Illumina MiSeq platform using 2 × 300 bp paired-end sequencing at the Genewiz sequencing facility (GENEWIZ Suzhou, China).

The microbial communities were analysed using QIIME 2 v2020.6 (Bolyen et al. 2019). The dereplicating of sequences and OTU (operational taxonomic unit) clustering at 97 % identity was done using the VSEARCH plugin (Rognes et al. 2016). Representative sequences for each OTU were assigned taxonomy using q2-feature-classifier (Bokulich et al. 2018) with the classifier pre-trained on GreenGenes v13.8 with 99 % OTUs. GreenGenes taxonomy was used provisionally (DeSantis et al. 2006; Balvociute and Huson 2017) up to the genus level; species level was not inferred from 16 S rRNA data. After quality filtering, 16 S rRNA gene amplicon data for 322 samples were included in the analysis with an average of 9560 reads per sample and a minimum of 1036 reads per sample. The sequence data is publicly available at the MG-RAST database under library accession number mgl837686 (https://www.mg-rast.org/).

### Statistical methods

All production data were analysed in SPSS v25 (IBM, Armonk, NY, USA) and significance was established at P < 0.05. Normally distributed data were analysed using a general linear mixed model. Generalised linear mixed models were applied to binary data (pregnancy and farrowing rate) using binary logistic regression and to count data (all piglet mortalities) using Poisson regression. Gestation and lactation periods were analysed as separate datasets. The model applied to the gestation data included gestation pen as a random term and treatment (CTR and PA) as a fixed effect. The model applied to lactation data included farrowing shed as a random term, and treatment (CTR, CTR-PA and PA) as a fixed effect. All data is expressed as mean ± standard error of the mean (SEM) unless it was binary data, whereby the confidence intervals are presented.

All of the downstream statistical microbial data analysis and visualisation were done using Calypso Version 8.84 (Zakrzewski et al. 2016) on a Hellinger transformed abundance table (Legendre and Gallagher 2001). Statistical analysis on alpha diversity metrics of Shannon’s index, Richness and Chao1 were performed. Multivariate data visualisations and multivariate statistical testing among treatment groups were performed using redundancy analysis (RDA), discriminatant anlaysis of principal components (DAPC) and Adonis analysis based on Bray-Curtis distance matrices. Univariate non-parametric Wilcoxon-rank tests were also applied to the data to identify the differences between specific taxa for each treatment. Core microbiota Venn diagram was also generated and plotted in Calypso Version 8.84 (Zakrzewski et al. 2016).

## Results

### Sow and litter performance

There was no effect of treatment on gestation weight gain (CTR: 59.3 ± 3.6, PA: 59.4 ± 3.6, *P* = 0.967) or P2 backfat gain (mm; CTR: 1.5 ± 0.6, PA: 1.2 ± 0.6, *P* = 0.411) during gestation. Pregnancy and farrowing rates were unaffected by gestation treatment (*P* > 0.05; Table 1). Litter size was increased by 0.8 pigs per litter in PA sows compared with CTR (*P* < 0.05; Table 1) however, this did not translate to a higher number of piglets born alive (*P* = 0.141) as stillbirths were higher in PA sows (*P* = 0.03). Additionally, the number of piglets born at less than 1.1 kg was significantly higher for PA sows (*P* = 0.015; Table 1).

**Table 1:** Reproductive performance of sows fed a control diet (CTR) or the control diet supplemented with a PA during the gestation period

	CTR	PA	*P-*value
Pregnancy rate (%)a	85.9 (79.9–90.4)	86.8 (80.5–91.2)	0.831
Farrowing rate (%)a	83 (76.4–88.1)	79.3 (72.0–85.0)	0.380
otal pigs born^b^	**12.7 ± 0.3**	**13.5 ± 0.3**	**0.034**
Total pigs born alive^b^	11.8 ± 0.2	12.3 ± 0.3	0.141
Total pigs born dead^b^	**0.90 ± 0.1**	**1.2 ± 0.1**	**0.030**
Day 1 average piglet weight (kg)^b^	**1.42 ± 0.04**	**1.34 ± 0.04**	**0.016**
Number of piglets less than 1.1 kg^b^	**3.6 ± 0.4**	**4.2 ± 0.5**	**0.015**

^a^Confidence intervals rather than SEM presented for binary data

^b^Data are expressed as mean ± SEM

Piglets were fostered to achieve the same litter size (11.7 ± 0.1 piglets per sow), but PA litters tended to exhibit a lower litter weight than CTR and CTR-PA post-foster (CTR: 16.8 ± 0.6, CTR-PA: 16.5 ± 0.6, PA: 15.7 ± 0.6, *P* = 0.080). There was no treatment effect on average daily gain (CTR: 0.215 ± 0.01, CTR-PA: 0.210 ± 0.02, PA: 0.214 ± 0.02, *P* = 0.797) and litter size (CTR: 10.1 ± 0.5, CTR-PA: 10.2 ± 0.5, PA: 10.0 ± 0.5, *P* = 0.713) or weight of piglets at weaning (day 21; CTR: 59.3 ± 5.1, CTR-PA: 60.3 ± 5.1, PA: 57.5 ± 5.1, *P* = 0.345).

There was no difference between treatments for pre-foster (CTR: 0.9 ± 0.2, CTR-PA: 0.7 ± 0.2, PA: 0.8 ± 0.2, *P* = 0.288), post-foster (CTR: 1.1 ± 0.1, CTR-PA: 1.0 ± 0.1, PA: 0.9 ± 0.1, *P* = 0.709) or total liveborn piglet mortality (CTR: 1.7 ± 0.4, CTR-PA: 1.4 ± 0.3, PA: 1.5 ± 0.4, *P* = 0.313). There were no treatment effects on sow feed intake, body weight or P2 backfat in lactation (*P* > 0.05; Table 2). There was a tendency for sows from the PA treatment to display the shortest rebreeding interval (*P* < 0.1).

**Table 2:** Weight, P2 backfat change in lactation, and wean to service interval of sows fed different dietary treatments (CTR, CTR-PA and PA)

	CTR	CTR-PA	PA	*P*-value
Average daily feed intake	7.1 ± 0.3	7.0 ± 0.3	7.2 ± 0.3	0.200
*Weight (kg)*				
Entry	280.1 ± 3.7	274.1 ± 3.7	277.9 ± 2.9	0.504
Exit	243.3 ± 9.4	237.6 ± 9.4	238.5 ± 9.2	0.443
Lactation change	− 36.6 ± 8.5	− 36.7 ± 8.5	− 38.6 ± 8.4	0.778
*Backfat thickness (mm)*				
Entry	19.1 ± 1.4	18.2 ± 1.4	18.2 ± 1.4	0.259
Exit	18.8 ± 0.1	18.8 ± 0.1	18.5 ± 0.1	0.221
Lactation change	− 0.7 ± 0.7	− 0.6 ± 0.7	− 0.4 ± 0.7	0.750
Wean to service interval (days)	9.0 ± 0.9	8.3 ± 0.9	6.4 ± 0.8	0.061

All data are expressed as mean ± SEM

### Impact of gestation diet on sow faecal microbiota

The administration of PAs to the gestation diet did not affect major alpha diversity metrics; Shannon’s index (*P* = 0.51), Chao1 (*P* = 0.46) and Richness (*P* = 0.59). Redundancy analysis (RDA) indicated a significant difference between the faecal microbiota of sows fed the CTR and PA diets in gestation (*P* = 0.001). Likewise, when assessing the microbiota structure differences using Adonis permutational multivariate analysis of variance based on the Bray-Curtis distance matrix, significant differences between the CTR and PA treatments existed (R^2^ = 0.02, *P* = 0.0003).

**Fig. 1:**
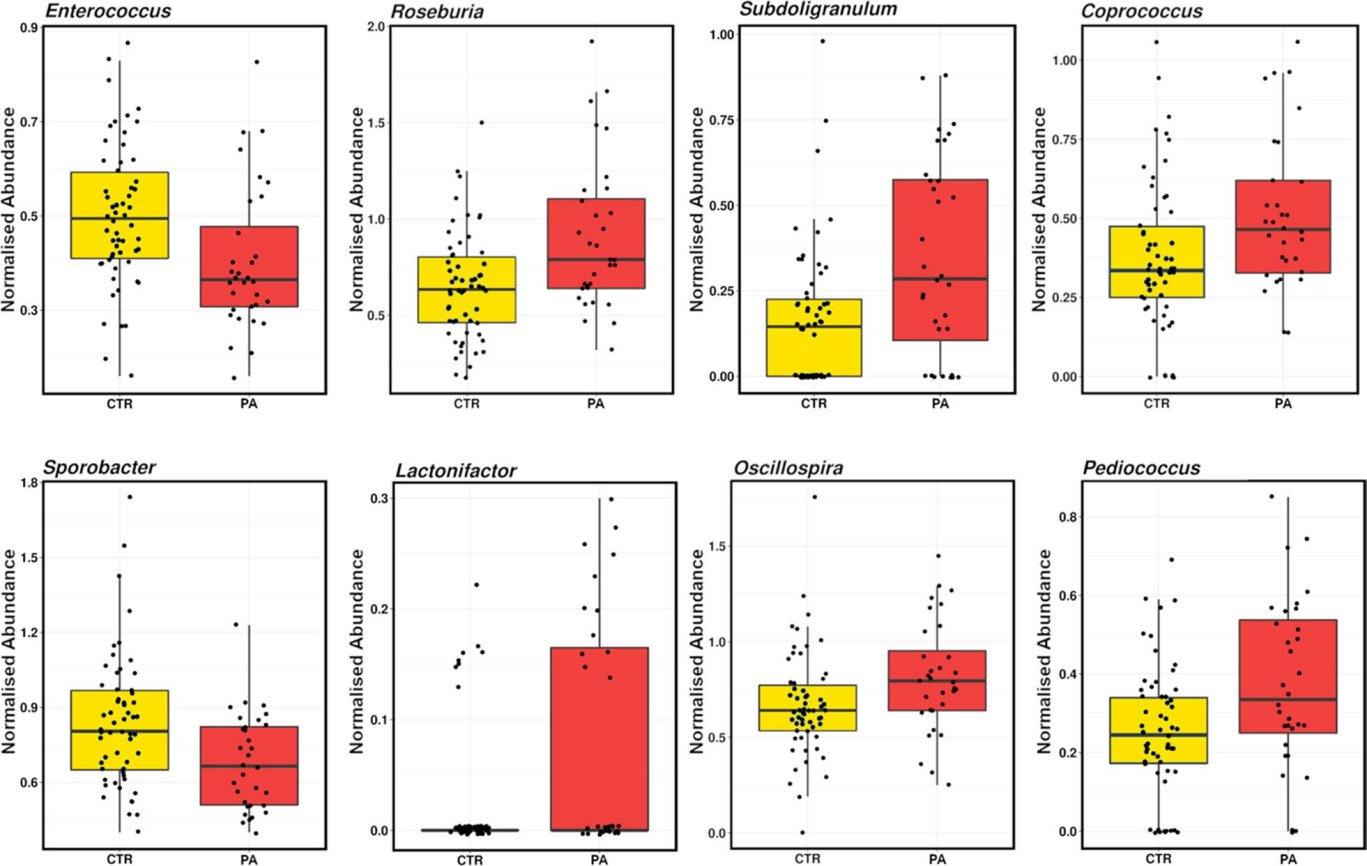
Classified genera significantly altered (*P* < 0.01) in the faeces of sows fed two different diets: a control diet (CTR), and a control diet supplemented with a PA during gestation

Differences in community structure were evident at the genus level, with 18 genera significantly affected by diet (Wilcoxon rank test; *P* < 0.05). Specifically, Unclassified p253418B5, Unclassified Bacteria, *Enterococcus, Sporobacter, Succinispira* and the archaea *Methanobrevibacter* were more abundant in control sows (CTR), while *Roseburia, Subdoligranulum, Lactonifactor, Oscillospira, Coprococcus, Pediococcus, p75a5, CF231, Prevotella, Ruminococcus*, Unclassified S247 and *Butyrivibrio* were more abundant in the faeces of PA sows. Those bacteria that contributed to *P* < 0.01 are presented in Fig. 1.

### Maternal influence on the core piglet microbiota at different ages

**Fig. 2:**
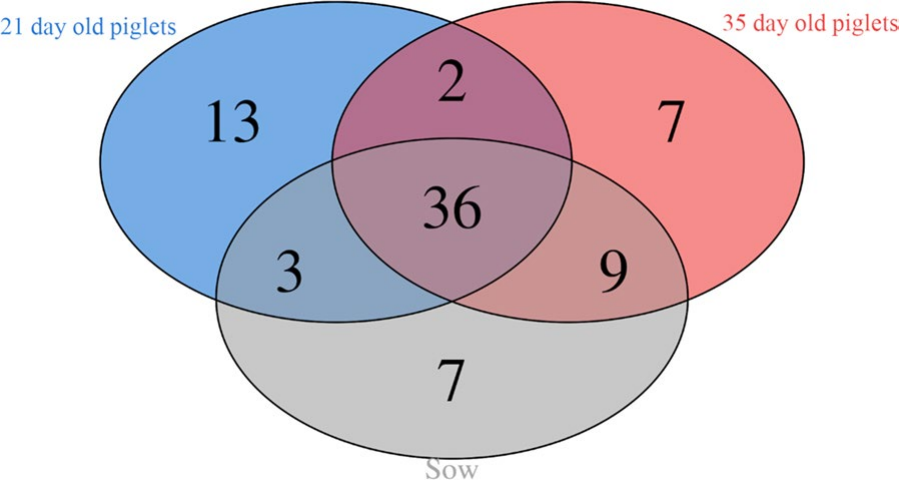
Venn diagram of core microbiota at the genus level between sows and piglets at 21 and 35 days of age

The influence of sow microbiota on the development and maturation of piglet intestinal microbial communities is presented in the Venn diagram depicting the core microbiota (Fig. 2). Of 77 total core genera, 36 (46 %) were core genera shared among sows, piglets at day 21, and piglets at day 35. Sows and piglets (including both day 21 and day 35) shared 62 % (48) of bacterial core genera, indicating the influence of maternal microbiota on piglets. A genus was considered a member of the group’s core microbiota if it was present in more than 40 % of the samples of that group.

### Impact of sow diet on piglet faecal microbiota

A significant shift in the microbial community occurred between day 21 and day 35 in piglets, moving their microbiota structure further away from the maternal influence; thus, we will present these separately.

### In 21-day-old piglets

**Fig. 3:**
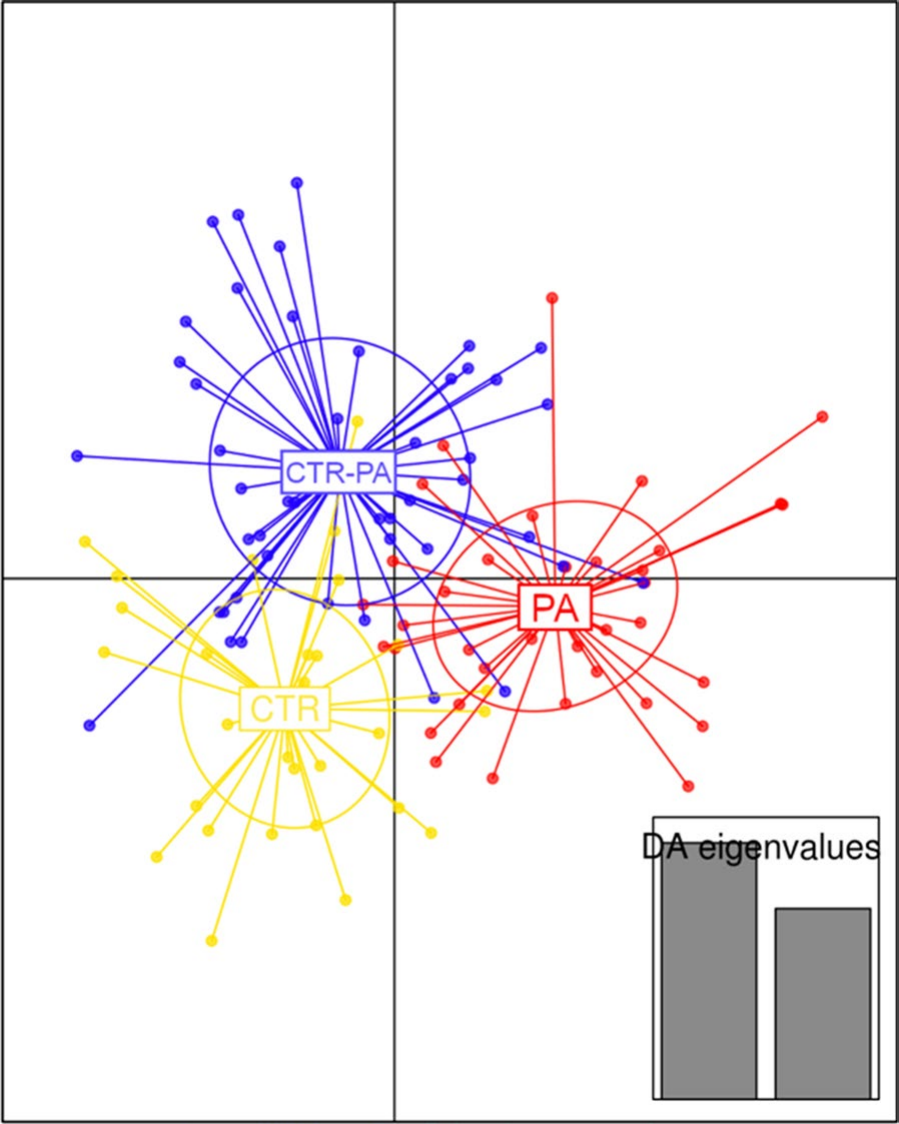
Discriminant analysis of principal components (DAPC) showing the relationship among 21-day old piglets reared on sows fed different dietary treatments (CTR, CTR-PA and PA). Each dot represents the microbiota profile from one piglet, while each ellipse represents the groups. Discriminant analysis (DA) eigenvalues of the analysis are displayed inset

In 21 d old piglets, there was no effect of sow diet on faecal alpha diversity measures (Shannon’s diversity, *P* = 0.48; Chao1, *P* = 0.38; and Richness, *P* = 0.88). A range of multivariate analyses and corresponding visualisation indicated some degree of overlapping occurred between treatments (CTR-PA, PA and CTR). Discriminant analysis of principal components (DAPC) showed that each treatment segregated from one another (Fig. 3). Additionally, Adonis permutational multivariate analysis of variance based on Bray-Curtis distance demonstrated a significant difference among the treatments (R^2^ = 0.02, *P* = 0.05).

**Fig. 4:**
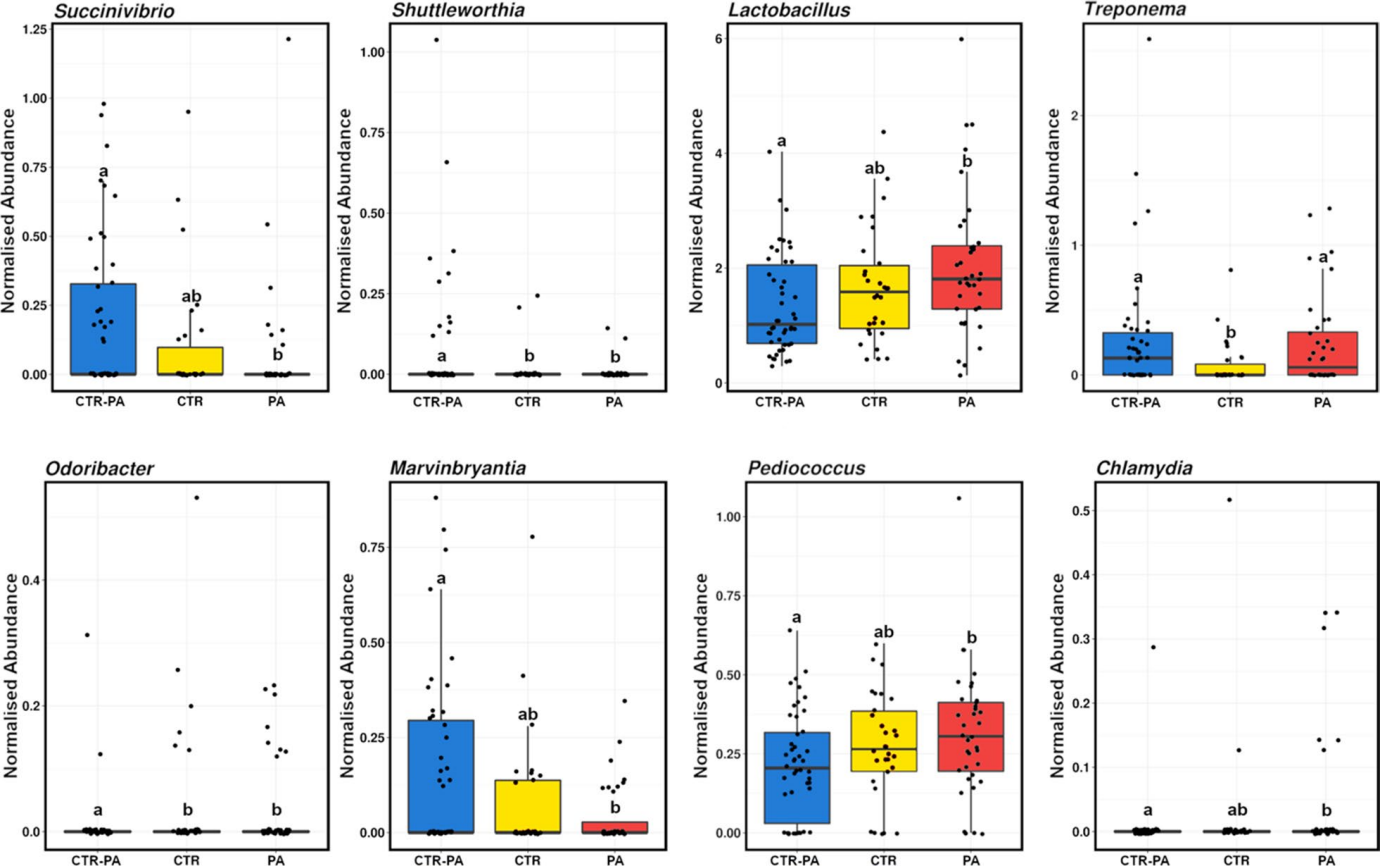
Genera significantly altered (*P* < 0.05) in the faeces of 21-day old piglets reared on sows fed different dietary treatments (CTR, CTR-PA and PA). Subscripts that differ denote a significant difference

Of the differences in community structure observed in the faeces of 21-day old piglets reared on sows fed differing diets, 8 genera differed significantly (Wilcoxon rank test; *P* < 0.05; Fig. 4). *Succinivibrio, Shuttleworthia*, and *Marvinbryantia* were most abundant in CTR-PA piglets, while *Treponema* were most abundant in CTR-PA and PA piglets, *Lactobacillus, Chlamydia* and *Pediococcus* were most abundant in PA piglets and *Odoribacter* were most abundant in CTR piglets.

### In 35-day-old piglets

**Fig. 5:**
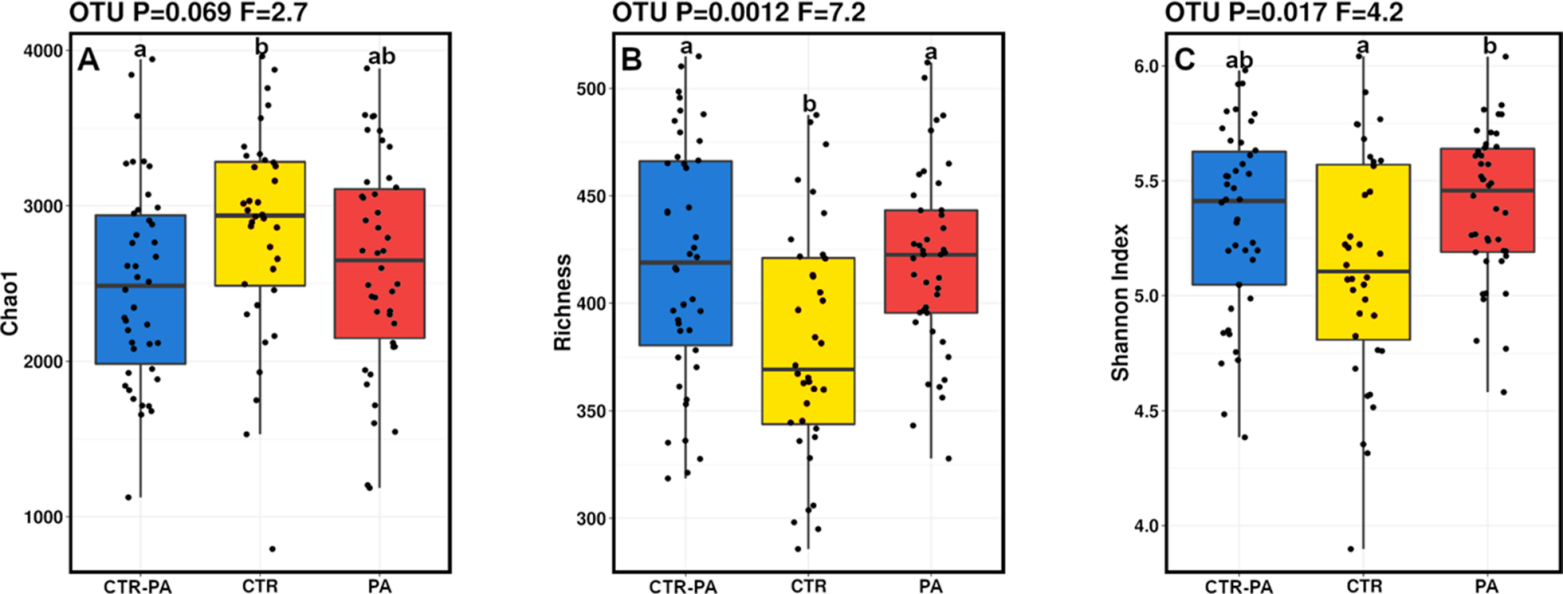
Boxplots demonstrating the change at genus level in **A** Chao1, **B** Richness and **C** Shannon’s diversity for 35-day old piglets that were reared on sows fed different dietary treatments (CTR, CTR-PA and PA). Subscripts that differ denote a significant difference

**Fig. 6:**
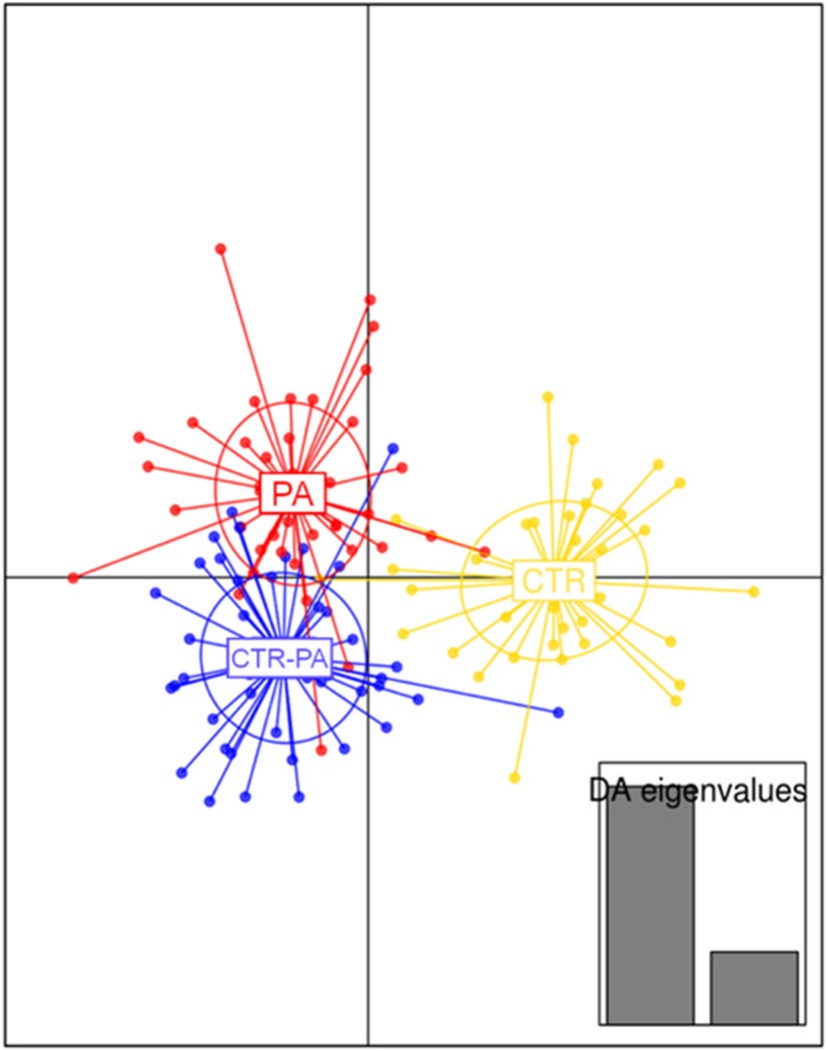
Discriminant analysis of principal components (DAPC) showing the relationships among 35-day old piglets reared on sows fed different dietary treatments (CTR, CTR-PA and PA). Each dot represents the microbiota profile from one piglet, while each ellipse represents the groups. Discriminant analysis (DA) eigenvalues of the analysis are displayed inset

Alpha diversity analysis showed that Shannon’s index (*P* = 0.02) and Richness (*P* = 0.001) were higher for those piglets reared on sows being fed PA, regardless of how long the sows received PAs for (PA and CTR-PA), while Chao1 tended to be higher for piglets reared on control sows (CTR; P = 0.07; Fig. 5). DAPC showed that piglets in the CTR-PA and PA treatment were more similar and clustered away from CTR piglets at 35-day of age (Fig. 6). Additionally, Adonis analysis based on Bray-Curtis distance matrices observed significant differences among treatments (R^2^ = 0.03, *P* = 0.002).

**Fig. 7:**
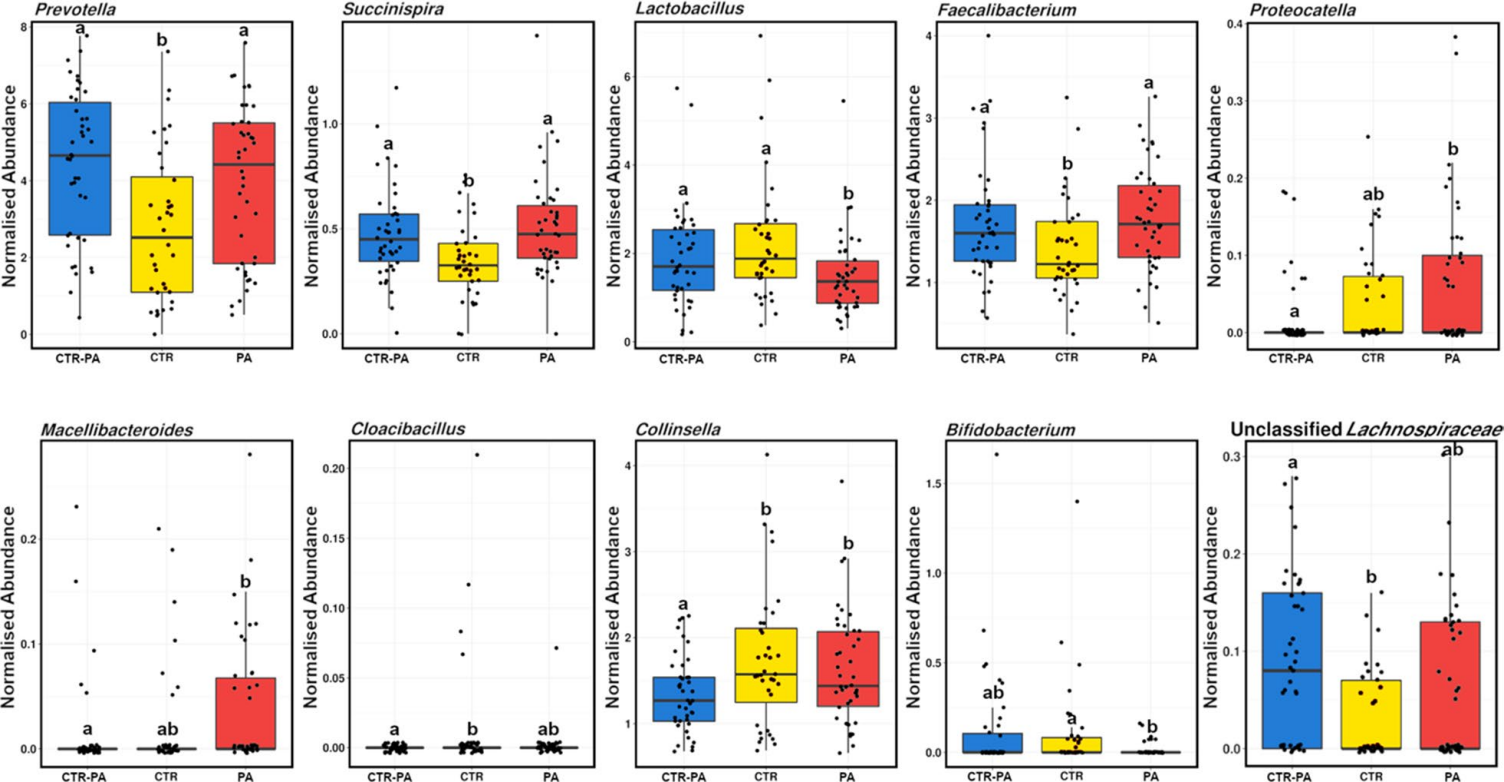
Genera significantly altered (*P* < 0.05) in the faeces of 35-day old piglets reared on sows fed different dietary treatments (CTR, CTR-PA and PA). Subscripts that differ denote a significant difference

There were 11 genera within the faeces of 35-day old piglets significantly affected by treatment, ten of which are presented in Fig. 7. Bacterial genera *Prevotella, Succinispira* and *Faecalibacterium* were most abundant in piglets reared on sows fed PAs regardless of the intervention length (CTR-PA and PA). *Lactobacillus* and *Bifidobacterium* were most abundant in piglets reared on sows fed a control diet throughout gestation (CTR-PA and CTR). *Proteocatella* and *Collinsella* were more abundant in CTR and PA piglets, while Unclassified *Lachnospiraceae* were more abundant in CTR-PA piglets. PA piglets had a higher abundance of *Macellibacteroides* and CTR piglets were more abundant in *Cloacibacillus* and archaea *Methanobrevibacter*.

## Discussion

### Sow gestation performance

Pregnancy is a time of high metabolic demand as fertilisation, implantation and embryo development occur. As a result, oxidative stress is a common by-product of these processes (Wang et al. 2018). Free-radical production is associated with many reproductive disorders (Berchieri-Ronchi et al. 2011) and therefore, a reduction in free-radicals would have positive implications for the sow. A key finding in the current study was an increase in litter size observed for those sows that were fed PAs in gestation. This increase in litter size has been documented previously in studies investigating the use of a different combination of PAs supplied during gestation (Reyes-Camacho et al. 2020). One possible explanation for how these additives influence litter size is their anti-inflamatory and antioxidative capacity. Supporting this notion, Reyes-Camacho et al. (2020) observed improvements in litter size and increased antioxidant enzyme activity as well as nitrous oxide levels during early gestation (d 35) when sows were fed PAs.

The PAs used may have caused an increase in litter size via two mechanisms. Although the essential oil components of the PA used are different from the study above, they may have effected litter size via their anti-inflammatory and antioxidant ability in the same way discussed above (Windisch et al. 2008; Karásková et al. 2016) or they may have influenced litter size via modulation of the GIT microbiota. Previous studies have identified specific bacteria associated with oxidative stress in sows (Wang et al. 2018, 2019). Wang et al. (2018) identified that antioxidant capacity was positively correlated with *Bacteroidaceae* but negatively with *Phascolarctobacterium* and *Streptococcus*, while Wang et al. (2019) reported correlations between *Ruminococcaceae* and *Coprococcus* with sows who gave birth to a higher number of stillborn piglets. In the present study, *Coprococcus* was increased in PA sows when compared to controls, and these animals had a significant increase in stillbirth rate, however, no other bacteria identified previously as being correlated with oxidative stress were observed. Additionally, sows that received PAs during gestation had a higher abundance of the potentially beneficial bacteria, *Oscillospira*, which is strongly correlated with the formation of secondary bile acids (Cheng et al. 2018) and *Roseburia* and *Ruminococcus*, known as butyrate-producing bacteria (Wang et al. 2018). Butyrate exerts a variety of functions that aid in maintaining GIT barrier function, it is an important energy source for colonocytes/epithelial cells, protects against inflammation and decreases oxidative stress, which can all lead to an improvement in feed efficiency (Hamer et al. 2007). Interestingly, CTR sow faeces were more abundant in genera *Enterococcus*, which has been associated with necrotising enterocolitis (Wang et al. 2016) and several *Enterococcus* species are associated with pathogenicity causing urinary tract infections, endocarditis and bacteremia (Singh et al. 2017). Additionally, PA sows had a higher abundance of potentially beneficial bacterial genera *Prevotella*, which has a unique ability to degrade mucin glycoproteins and increase weight and survival in pigs (McCormack et al. 2017; Wang et al. 2017). However, Wang et al. (2018) has demonstrated that it is correlated with 8-hydroxy-deoxyguanosine which is a marker for oxidative damage in sows. Together, the results suggest that PA may be beneficial by reducing potentially pathogenic *Enterococcus* and enhancing butyrate-producing bacteria and hence improve intestinal barrier function, decreasing oxidative stress. However, further research is needed to assess these effects directly.

### Sow lactation performance

Whilst the total number of piglets born increased in the PA treated sows, this failed to translate to an increase in the number of piglets born alive. This contrasts with other published studies with PAs that utilised a similar experimental design (Reyes-Camacho et al. 2020). An increase in the number of piglets born dead in the PA treated group was observed, which likely explains why no improvement in born alive was observed. However, no autopsy was completed on dead piglets, and rather piglets were classified as dead at birth by the presence of caps on feet (i.e., they had not walked). The sows farrowed in naturally ventilated rooms throughout the trial and the average minimum temperature was 6 °C (maximum 16 °C), and except for creep heat lamps, no additional heat was provided in the farrowing shed. The 60 g reduction in average birth weight in piglets from PA sows, likely due to the increased litter size in this group, also increases the probability that these piglets died from exposure, as low-birth-weight piglets are naturally at a higher risk of mortality (Baxter et al. 2008). Taken collectively, the reduced birthweight in PA piglets and the low ambient temperature during the experimental period may have increased the risk of deaths from exposure which were incorrectly categorised as stillbirths. Thus, these piglets might have survived if the farrowing room environment was optimised.

There was no impact of the PA on lactation sow feed intake, litter weight or sow body condition at weaning. Surprisingly, there was a tendency for a 2 to 3-day reduction in the interval from weaning to breeding. It is unknown why this improvement in reproductive performance was observed in the absence of significant changes in total feed intake and body condition. Presumably, it involves a positive effect on ovarian follicular growth. Others have postulated that the anti-inflammatory and antioxidant properties of the phytogenics containing oregano fed around the time of farrowing improve uterine involution, and this is what leads to the reduction in wean to service interval (Kis and Bilkei 2003). Regardless, this finding has implications for non-productive days in sow herds and potentially for subsequent fertility.

### Piglet performance

It is well established that the development of the GIT microbiota is important for health and survival in all species. The farrowing house provides the first place to influence the development of the microbiota of the piglet as the piglet is housed exclusively with their sow. It is well understood in commercial operations that the sow’s microbiota can have positive and negative impacts on the piglet if not well managed. Finding that PA fed sows, regardless of whether it was fed in gestation and/or lactation or just lactation, altered the microbiota of piglets at 21 days of age was somewhat expected but has not been widely demonstrated. Previous research suggests that the GIT microbiota develops rapidly during early lactation and is influenced by a combination of factors, including the sow’s urogenital microbiota, colostrum and milk consumption, the pen microbiota, and interaction with the sow’s faeces (Nowland et al. 2019). Therefore, it is likely that the piglet’s microbiota was modulated via one or more of these processes. This is further substantiated by the finding that sows shared 62 % of core genera with their piglets in the present study. Additionally, previous research investigating the use of PAs in sow diets throughout gestation or gestation and lactation demonstrated that phytogenic volatile compounds were present in the placental fluid of those animals fed the additive throughout gestation and were present in the milk of those fed the additive throughout lactation (Reyes-Camacho et al. 2020). Hence, it is possible that GIT modulation was initiated before parturition in the PA piglets and persisted throughout lactation from its presence in the milk in the PA and CTR-PA sows. Additionally, piglets have been known to exhibit coprophagy and hence it would be expected that this would have contributed to the change observed (Aviles-Rosa et al. 2019).

Although the faecal microbiota of piglets was altered by the inclusion of PAs in sow diets, no improvements in production parameters such as piglet weight and survival were observed. This contrasts with a previous study where PA fed grower-finisher pigs demonstrated improvements in growth (Walker et al. 2019). However, dosage may have affected this outcome as the PA concentration in milk is likely lower than what they would have received in the feed. Additionally, a milk fed animal is very different from one on solid feed and hence this may have also had an impact. When investigating the faecal microbiota at 21 days of age, a combination of potentially beneficial and potentially pathogenic bacteria were present in piglets reared on PA and CTR-PA sows. 21-day-old PA piglets were more abundant in *Lactobacillus*, which is known for its probiotic attributes, being associated with improved GIT health, feed efficiency and growth in pigs (Shu et al. 2001). While *Chlamydia*, a potentially pathogenic bacteria, was also more abundant in PA piglets at 21 days of age. Additionally, *Treponema*, a potentially pathogenic bacterial genus, previously associated with swine dysentery (Rees et al. 1989) was more abundant in piglets reared on CTR-PA and PA sows. These results suggest that although the faecal microbiota of 21-day old piglets was influenced by PAs, no apparent advantage or disadvantage for piglet growth performance was evident.

Interestingly, differences in faecal microbiota between piglets reared on PA and CTR-PA sows when compared with CTR animals existed two weeks post-weaning (d35) even when the influence of the sow was removed. Additionally, the faecal microbiota of piglets from sows fed PAs also tended to cluster closer together and become more similar postweaning. Weaning is a time of high stress and can cause postweaning diarrhoea and often results in a postweaning growth check (Pluske et al. 2018). Therefore, the presence of an “optimal” microbiota during this time may be beneficial. Unfortunately, no post-weaning pig weights could be collected on these pigs, so no assessment of piglet productivity occurred. Regardless, piglets reared on sows fed PAs were colonised by multiple potentially beneficial bacteria postweaning. At 35 d, PA and CTR-PA piglets had an increased abundance of *Faecalibacterium*, which is a butyrate-producing bacteria with anti-inflammatory effects (Singh et al. 2017), and a short chain fatty acid producing bacteria, *Succinispira* (Janssen and O’Farrell 1999). While potentially beneficial bacteria, *Prevotella* and *Bifidobacterium*, which are positively correlated with body weight (Shu et al. 2001; McCormack et al. 2017) and likely butyrate-producing bacteria, Unclassified *Lachnospiraceae* (Cheng et al. 2018), were assessed as explaining some of the microbial differences between piglets and were most abundant in CTR-PA 35-day old piglets. This indicates potentially improved intestinal health and an associated growth in these animals. Additionally, CTR-PA and CTR piglets shared a higher abundance of potentially beneficial bacteria, *Bifidobacterium* and *Lactobacillus*, at 35-days of age. The 35-day old CTR piglets had a higher abundance of the potentially pathogenic bacteria, *Cloacibacillus*, which is a potential human pathogen associated with bacteremia (Domingo et al. 2015). Overall, without the added production characteristics it is difficult to distinguish whether the PA provided any benefit to the piglets. This study provides evidence that microbiota manipulation of the sow influences the piglet microbiota and that this influence persists for at least two weeks beyond weaning.

Our findings demonstrate that PAs altered the microbiota of sows and that this change was transferred to their piglets and was maintained for up to 14 days post-weaning. Additionally, the inclusion of PAs to a gestation diet increased the number of piglets born, presumably via its antioxidant effects, however, this was not evident as liveborn piglets. While no further improvements in weight or survival parameters were observed in the sows and piglets during lactation, the wean to oestrus interval tended to be reduced in sows fed the PA throughout gestation and lactation. Therefore, the inclusion of PAs in a sow diet throughout gestation and lactation has the potential to increase the number of piglets born per sow and reduce the number of non-productive days. Further research investigating how PAs influence litter size and what effect it is having on the GIT microbiota of piglets reared post-weaning in relation to performance parameters is warranted.

## Supplementary Information

**Additional file 1: Table S1. **Gestation and Lactation base diet specifications.

## Data Availability

Sequencing data is publicly available on MG-RAST metagenomic data server database (https://www.mg-rast.org/) under library accession number mgl837686.
