# Supplementary material for: Maternal supplementation with phytogenic additives influenced the faecal microbiota and reproductive potential in sows
Source: AMB Express. 2021 Jul 15;11:107. doi: 10.1186/s13568-021-01268-8 (PMC8282833; doi:10.1186/s13568-021-01268-8)
Supplement: Supplementary file 1 — Additional file 1: Table S1. Gestation and Lactation base diet specifications. [file 13568_2021_1268_MOESM1_ESM.docx]

**Supplementary table 1.** Gestation and Lactation base diet specifications.

|  | **Gestation** | **Lactation** |
| --- | --- | --- |
| Barley % | 59.6 | 24 |
| Wheat % | 19.2 | 40.8 |
| Millrun % | 8.2 | 5 |
| Peas % | - | 10.3 |
| Canola meal % | 3 | - |
| Soybean meal % | - | 3.4 |
| Meat meal % | 2.5 | 5.4 |
| Blood meal % | - | 0.5 |
| Vegetable oil blend % | 1.4 | 3 |
| Salmon oil % | - | 0.4 |
| Limestone % | 1.2 | 0.8 |
| DE MJ/kg | 13 | 14 |
| Protein % | 13.1 | 17.3 |
| Calcium % | 0.9 | 0.97 |
| Phosphorus % | 0.6 | 0.64 |
| SID Lysine % | 0.55 | 0.84 |
| Methionine % | 0.3 | 0.34 |
| Threonine % | 0.55 | 0.67 |
| Tryptophan % | 0.15 | 0.2 |
| Isoleucine % | 0.47 | 0.62 |
| Valine % | 0.63 | 0.82 |
| Leucine % | - | 1.18 |
